# On the development of gestural organization: A cross-sectional study of vowel-to-vowel anticipatory coarticulation

**DOI:** 10.1371/journal.pone.0203562

**Published:** 2018-09-14

**Authors:** Elina Rubertus, Aude Noiray

**Affiliations:** 1 Department of Linguistics, University of Potsdam, Potsdam, Germany; 2 Haskins Laboratories, New Haven, Connecticut, United States of America; University of Birmingham, UNITED KINGDOM

## Abstract

In the first years of life, children differ greatly from adults in the temporal organization of their speech gestures in fluent language production. However, dissent remains as to the maturational direction of such organization. The present study sheds new light on this process by tracking the development of anticipatory vowel-to-vowel coarticulation in a cross-sectional investigation of 62 German children (from 3.5 to 7 years of age) and 13 adults. It focuses on gestures of the tongue, a complex organ whose spatiotemporal control is indispensable for speech production. The goal of the study was threefold: 1) investigate whether children as well as adults initiate the articulation for a target vowel in advance of its acoustic onset, 2) test if the identity of the intervocalic consonant matters and finally, 3) describe age-related developments of these lingual coarticulatory patterns. To achieve this goal, ultrasound tongue imaging was used to record lingual movements and quantify changes in coarticulation degree as a function of consonantal context and age. Results from linear mixed effects models indicate that like adults, children initiate vowels’ lingual gestures well ahead of their acoustic onset. Second, while the identity of the intervocalic consonant affects the degree of vocalic anticipation in adults, it does not in children at any age. Finally, the degree of vowel-to-vowel coarticulation is significantly higher in all cohorts of children than in adults. However, among children, a developmental decrease of vocalic coarticulation is only found for sequences including the alveolar stop /d/ which requires finer spatiotemporal coordination of the tongue’s subparts compared to labial and velar stops. Altogether, results suggest greater gestural overlap in child than in adult speech and support the view of a non-uniform and protracted maturation of lingual coarticulation calling for thorough considerations of the articulatory intricacies from which subtle developmental differences may originate.

## Introduction

In spoken language, speech segments overlap with each other. These coarticulatory effects have been detected in the acoustic output of speech as well as in the shapes, positions, and movements of the active articulators of speech, the lips, the tongue, the velum, and the larynx (for a review see Hardcastle & Hewlett [1]). The present study focuses on lingual coarticulatory processes and aims to outline their maturation across childhood. In adults, the positioning and shaping of the tongue is not only determined by the segment currently under production but shows characteristics of neighboring speech segments at the same time. These gestural overlaps exist in heterorganic sequences employing different articulators for achieving the consonantal and vocalic gestures (e.g., /ba/ where the tongue body anticipates a low back position for /a/ during the production of /b/) as well as in homorganic sequences involving the same primary articulator for both gestures (e.g., the point of contact between the tongue body and the palate or velum for /g/ varies with the frontness of the following vowel) (e.g., [2]). The domain a vowel can influence this way is not restricted to its adjacent neighbors but can span several segments in an utterance (e.g., [3]). While a still growing body of literature has described adults’ lingual anticipatory processes extensively, similar scrutiny for the maturation of this organizational scheme in childhood has lacked. Most developmental studies have focused on coarticulatory processes within the syllabic domain (intrasyllabic coarticulation; e.g., [4–8]). Yet, research on intersyllabic processes is crucial because it tackles a broader organization of speech production processes and therefore addresses questions about the interplay between cognitive (e.g., phonological planning, gestural phasing) and motor domains (the physical implementation).

To begin to fill this gap, the present study tracked the maturation of vowel-to-vowel (V-to-V) coarticulation in four groups of German children (from 3 to 7 years of age) in comparison to adults. Before presenting our data, we first briefly review existing evidence of V-to-V coarticulation in adults as well as suggested implications for planning and motor processes and provide an overview of the existing body of literature in the developmental field that the present study aims to augment. Finally, we relate our findings to previous literature and discuss whether the outcome pattern could be explained by differences between children’s and adult’s gestural organization.

### Vowel-to-vowel coarticulation in adults

Adults begin to produce the vowel for a forthcoming syllable during a preceding syllable. Multiple studies have provided evidence for coarticulatory effects of V_2_ in the domain of the transconsonantal vowel V_1_ in vowel_1_-consonant-vowel_2_ (V_1_CV_2_) sequences. This lingual anticipation has been either measured in the acoustic signal by comparing formant values (e.g., [9–13]) or (additionally) in the articulatory signal by directly observing changes in tongue positioning (e.g., x-ray: [14,15]; electropalatography: [16,17]; electromagnetic articulography: [18]). The magnitude of these V-to-V coarticulatory effects however, was shown to vary with several factors: Among others, data from Beddor, Harnsberger, & Lindemann [12] and Manuel [19] suggest a language dependency according to which vowels from dense inventories are anticipated to a lower degree than those from relatively sparse inventories. Suprasegmental factors also impact on the degree of vocalic anticipation with stressed vowels being less affected by contextual effects than unstressed vowels [10]. One of the main characteristics of unstressed vowels being a reduction of articulatory strength approaching schwa (e.g., [20]), it follows logically that schwa is more malleable and therefore affected to a higher degree by coarticulatory processes than full vowels (for a discussion, see Browman & Goldstein [15]).

#### The role of the intervocalic consonant

Finally, another influencing factor of particular interest for the current study is the nature of the intervocalic consonant. In measures of vowel anticipation during the preceding consonant itself (i.e., V-to-C-coarticulation), there are consistent effects of a consonant-specific property [2,21,22]: “coarticulatory resistance” [23] refers to how likely a segment’s articulatory gestures are to be coproduced with those of another. As conceptualized in the Degree of Articulatory Constraints model (DAC), the more the tongue dorsum is constrained during the production of a segment, the less likely this segment is to coarticulate with its neighbors [24]. Accordingly, labial consonants were shown to display lower coarticulatory resistance and therefore more lingual coarticulation with following vowels than alveolar consonants [22,25,26]. Palatal consonants like /ɲ/ on the other hand, put more constraints on the tongue dorsum and were found to be even more resistant to vocalic influences than alveolar consonants (e.g., [21]). However, velar consonants like /g/ display a rather low coarticulatory resistance despite of employing the tongue dorsum [2], because the location of tongue body contact with the palate is relatively flexible [27]. Consequently, /g/’s exact place of articulation usually varies along the front-back dimension according to its vocalic context.

This differing permeability of consonants can be attributed to mechanisms ensuring the achievement of phonetic targets and their intelligibility. However, whether those mechanisms are implemented in the speech production system at a rather early level adjusting the speech plan with regard to contextual variation (e.g., look-ahead models / feature-spreading models) or at a later stage of physical implementation in the vocal tract (e.g., coproduction models) is a matter of dispute (cf. [28] for overview and discussion). While the former theories assume variable gestural plans (e.g., [29,30]), the latter build on temporally invariant underlying gestures (e.g., [18,31]).

Expanding the concept of coarticulatory resistance and context sensitivity to V_1_CV_2_ sequences, one could hypothesize high resistant consonants to also limit V_2_’s influence on V_1_. Indeed, among others, Recasens [11,16], and Fowler & Brancazio [18] found influences of the intervocalic consonant’s resistance on the degree of V-to-V coarticulation. However, in none of these three studies results were entirely consistent. First, within the rather limited sets of participants, there were some speakers whose V-to-V coarticulation was not at all affected by the intervocalic consonant’s resistance. And second, instances of V_1_CV_2_ sequences were found that indicated anticipatory V-to-V coarticulatory effects but at the same time no V-to-C effects [16,18]. Despite high resistant consonants’ articulation not being affected by the vocalic gestures themselves, they did thus not always attenuate V_2_’s influence on the preceding vowels. These occasional findings of discontinuous coarticulatory effects were interpreted as evidence for a speech production model assuming gestural plans of relatively invariant phasing and activation curves to be combined and coproduced in fluent speech [18,31]. According to Fowler & Saltzman [31], it is implausible for these discontinuous effects to be part of a speech plan because there is no reason to start, stop, and restart producing a vocalic gesture. Within the coproduction framework the sequencing of consecutive gestures in the planning phase of an utterance is predetermined and quasi blind to contextual variations. Consequently, coarticulatory effects are not part of the speech plan (as contrarily suggested by Whalen [30]) but occur only during the physical implementation of the gestures in the vocal tract.

Taken together, the literature on V-to-V lingual coarticulation in adults shows that vowels are initiated already during the production of preceding segments. The strength of this vocalic anticipation seems to depend on several factors, one of which is the coarticulatory resistance of the intervocalic consonant. How and in which conditions exactly the consonant’s resistance modulates V-to-V coarticulation, however, is not consistently deducible from existing studies yet.

### Coarticulatory processes in children

#### Intrasyllabic coarticulation

Turning to the maturation of coarticulatory processes in children’s speech, previous studies have almost exclusively focused on measures of intrasyllabic V-to-C coarticulation. The overarching aim of most of these studies was to infer the unit size of gestural organization and control at different ages. While a low degree of coarticulation between consecutive segments is in that respect interpreted to indicate a segment-driven language organization, a high degree of coarticulation suggests control units larger than the segment. However, diverging results were found: An increasing or stable coarticulation degree across age in some studies (e.g., [32–34]) as well as a decreasing coarticulation degree with age in other studies (e.g., [4,35–37]). Hence, there is a large discrepancy in the theoretical propositions of researchers ranging from theories suggesting that organizational units grow from the size of a segment to (at least) syllable size with age and language experience, to views suggesting a reduction of unit size with language development such that children initially organize their speech in broad (possibly syllabic) units and develop finer and more differentiated control for single segments only later.

In previous analyses of the present sample of German participants, we noted a decrease of intrasyllabic coarticulation degree from 3 years of age to adulthood [37]. This finding raised the question whether vocalic anticipation in young children extends beyond the syllabic domain. Furthermore, we found consistent effects of the consonant’s coarticulatory resistance on the degree of V-to-C-coarticulation with the vowel’s tongue position being anticipated most during /b/, to an intermediate degree during /g/, and least during the production of /d/. This result provided a main incentive for the present investigation of consonant-related differences in intersyllabic coarticulation effects.

#### Intersyllabic coarticulation

In the literature addressing coarticulation across syllable boundaries in child speech, findings are as inconsistent as they are for intrasyllabic coarticulation. The early studies measured second formant frequencies in syllable-final schwas followed by a syllable with a full vowel nucleus [38,39]. Repp [38] reported V-to-V coarticulation from the full vowel to the preceding schwa in an English-speaking adult as well as in his 9-year-old participant but not in his 4-year-old participant. In a more extensive study of 10 participants per age cohort, Hodge [39] reached similar results with an age-related increase in coarticulatory degree from vowels to preceding schwas in “a stee” and “a stew” utterances: 3-year-olds showed a non-significant trend towards V-to-V coarticulation, and 5-year-olds anticipated the upcoming vowel to a lesser degree than 9-year-olds who in turn exhibited less coarticulation than adults. While these results suggest that V-to-V coarticulation becomes stronger with age, other studies provided evidence that young children already exhibited a magnitude of V-to-V coarticulation similar to that of adults: 3-, 5-, and 7-year old children and adults displayed significant effects of the vowel’s second formant frequency on that of schwa in English schwa-C-V (əCV) sequences [4,40]. The magnitude of this V-to-V coarticulation did not vary with age. Interestingly, across Nittrouer’s [40] whole data set the effect of the vowel on the schwa interacted with the factor stop consonant identity. Expanding Recasens’ [11,16] and Fowler & Brancazio’s [18] findings, her results therefore provide evidence for vowel anticipation during schwa to be stronger in /k/ contexts than in /t/ contexts. In a longitudinal study, Goodell & Studdert-Kennedy compared acoustic coarticulatory effects of different segments in English CəCV sequences between children at 22 and 32 months of age and adults [41]. While the absolute formant values suggested a decrease of anticipatory V-to-V coarticulation with age, after a normalization procedure accounting for the differences in vocal tract size, group differences disappeared for utterances ending in /i/, and for those ending in /a/ only 22-month-olds remained to show significantly greater V-to-V coarticulation than 32-month-olds and adults.

Contradicting these findings, there is also evidence that children show stronger acoustic effects of vowel anticipatory coarticulation than adults do: In a study comparing typical to atypical speech production development in Dutch, the typically developing 5- to 7-year-olds exhibited stronger V-to-V coarticulation than the adult control group [35]. Similar to the previously reported studies, they looked at measures of schwa’s second formant in əCV utterances. The hypothesis that this pattern could be specific to the Dutch language, is called into question by another study on English-speaking children providing evidence for stronger V-to-V coarticulation in 3-, 4-, and 5-year-olds than in adults as measured in first and second formant frequencies of English əCV sequences [42].

All developmental studies reported so far have employed acoustic measurements of lingual V-to-V coarticulation. While articulatory data can provide more direct insights into speech production mechanisms, most articulatory data collection techniques are not suitable for young children due to their invasiveness (e.g., articulography, MRI). In the last two decades however, ultrasound imaging has become a popular method for observing and collecting tongue data in the young age (e.g., in kindergarten: [7,37,43]; in toddlers: [44]). Barbier and colleagues [45] report on one of the few studies investigating the maturation of long-distance coarticulation with articulatory in addition to acoustic measurements. They compared Canadian French 4-year-old’s articulation of VCV sequences to that of adults. While significant lingual V-to-V coarticulation was observed in adult speakers, only some of the children exhibited vocalic anticipation. The authors therefore concluded that as a group, children were unable to anticipate a vowel’s tongue configuration during the production of transconsonantal vowels. It should be noted however, that contrary to the previous studies, they did not investigate the vowel’s effect on a preceding schwa but on full vowels (/ε/ and /a/).

In summary, while most studies showed that children anticipate a vowel during a preceding schwa at least to some extent, there is conflicting evidence for all three possible scenarios of the V-to-V coarticulation degree’s development: A decrease with age, an increase with age, or a similar coarticulation degree throughout development. Several reasons may (in part) explain the discrepancies in results found for both intrasyllabic and intersyllabic coarticulation. First, decisions about the design of the study such as the utterance type and the data collection technique (e.g., method, measurement time point) might be a source of contradiction. In addition, a shortcoming of especially the early studies is the very limited number of participants and its impact on statistical power. Given the fast and multi-faceted developments taking place in the anatomical, cognitive, and speech motor control domains during childhood, the speech of children is known to be highly variable both within and between speakers. It is therefore important to investigate large samples of children and narrow the age range within a cohort to a minimum.

### Goal and research questions

The overarching goal of this study is to uncover the development of V-to-V coarticulation in German children. In combination with other studies within our research agenda, we aim to provide insights into the underlying mechanisms of typical speech production to be used for diagnostic and potentially therapeutic purposes among German children with speech impairments. We hope to overcome some of the restrictions of previous research outlined above by: a) Investigating four larger age cohorts across childhood and one cohort of adults. Each age cohort includes at least 13 participants within a narrow age range to minimize age-related variability within the cohorts and therefore increase statistical power. b) Employing a well-controlled set of stimuli varying in place of articulation to investigate differences in coarticulatory degree between phonetic contexts. c) Recording speech material with ultrasound tongue imaging, a non-invasive technique allowing for direct access to tongue positions rather than their estimation via acoustic measures.

To assess whether children differ from adults in how strong vocalic gestures are activated and coproduced with a preceding schwa, measures of the vowel-related change in the horizontal position of the highest point of the tongue body during schwa in schwa-C-V sequences were analyzed according to the following three research questions: First, do we observe anticipatory V-to-V coarticulation in children of every age investigated as well as in adults? If the horizontal tongue body position during schwa varies as a function of tongue position during the following vowel, it will provide evidence for anticipatory V-to-V coarticulation. Although its magnitude varied tremendously in previous studies, evidence for anticipatory V-to-V coarticulation in children was found in most studies. We therefore expect every cohort to anticipate the upcoming vowel during schwa. Second, is the degree of anticipatory V-to-V coarticulation modulated by the resistance of the intervocalic consonant? If so, less V-to-V coarticulation should be found in cases in which consonantal resistance is higher (i.e., alveolar context) than when resistance is lower (i.e., labial context). However, predictions are hard to formulate because this question has been addressed only sparsely in adults providing complicated outcome patterns [11,16,18] and was only investigated on the margins for children so far [40]. Based on Nittrouer’s [40] findings, we expect a higher degree of V-to-V coarticulation in sequences with low resistant intervocalic consonants (here /b/ and /g/) than in sequences with high resistant consonants (here /d/). The flexibility of the place of palate contact for /g/ might trigger vowel-related fronting or backing of the tongue during schwa resulting in a high (but ‘mediated’) V-to-V coarticulation degree. And third, are there developmental changes in terms of coarticulation degree and consonantal effects? This question will be addressed by investigating differences between age cohorts. Again, the conflicting results of previous investigations prevent a clear formulation of predictions. Yet, the considerable decrease of V-to-C-coarticulation degree with age in the previous analysis of this data corpus [37] leads us to predict the same direction for the current investigation of V-to-V coarticulation.

## Method

### Participants

In total, 75 participants of five different age cohorts were recorded: 19 3-year-old children (10 females, age range: 3;05–3;09 (Y;MM), mean: 3;06), 14 4-year-old children (7 females, age range: 4;04–4;08, mean: 4;05), 14 5-year-old children (7 females, age range: 5;04–5;07, mean: 5;06), and 15 7-year-old children at the end of their first or beginning of their second grade in primary school (10 females, age range: 7;00–7;06, mean: 7;02). The adult cohort included 13 adults (7 females, age range: 19–28 years, mean: 23). All participants were from monolingual German families and none of them reported any language-related, hearing-related, or visual problems. Adult participants as well as the parents of the child participants gave written informed consent for participation in the study and all were provided with the option to stop participation at any time without negative consequences. The study was approved by the Ethic Committee of the University of Potsdam.

### Stimulus material

Trochaic pseudowords of the form consonant_1_-vowel-consonant_2_-schwa (C_1_VC_2_ǝ) that were recorded by a native German female adult speaker served as model stimuli for a repetition task. The consonants used in both positions were /b/, /d/, and /g/. The three places of articulation were chosen because they vary in coarticulatory resistance. The vowel set consisted of the tense and long vowels /i/, /y/, /u/, /a/, /e/, and /o/ which represent the German vowel space quite adequately. C_1_Vs were designed as a fully crossed set of Cs and Vs to which the second syllable was added, C_2_ was never the same as C_1_. These pseudowords were embedded in a carrier phrase with the German female article /aɪnə/ resulting in utterances such as for example /aɪnə bi:də/. Anticipatory V-to-V coarticulation was measured between the full vowel of the pseudoword and the preceding schwa in the article.

The total number of trials per child varied with group because 4- and 7-year-olds’ stimulus sets included the additional C_1_ /z/ which is not analyzed here. Repeating every word 3 times, 3- and 5-year-olds ended up with 108 trials and 4- and 7-year-olds with 138 trials. For all cohorts of children, trials were presented in 6 semi-randomized blocks. Adults’ stimulus set included /z/ in both consonant positions adding to a total number of 216 trials, which were presented in 9 randomized blocks. Mispronounced trials were noted down by the experimenters and if possible repeated at the end of the block. A table summarizing the number of trials used for the present analyses per consonant context per age cohort is provided in S1 Table.

### Experimental procedure

All recordings took place at the Laboratory for Oral Language Acquisition (LOLA) at University of Potsdam (Germany). Participants were asked to repeat a series of pre-recorded auditorily presented stimuli while they were recorded within the SOLLAR-platform (Sonographic and Optical Linguo-Labial Articulation Recording system [46]). This child-friendly setup allows for simultaneous recordings of tongue motion using ultrasound imaging (Sonosite, sr.: 48Hz), labial movement via video recording (camera SONY, sr.: 50Hz) and the audio speech signal (microphone Shure, sr.: 48kHz). For the recording, adult participants sat in a comfortable chair and children in a car-seat adjustable in height. The ultrasound probe was positioned straight below the participant’s chin between the maxillary bones to record the tongue surface contour in the midsagittal plane. It is fixed on a custom-made probe holder to be flexible in the vertical dimension following natural speech-related vertical jaw movements but prevents motion in lateral and horizontal translations. Additional head-to-probe stabilization was not employed to maximize the naturalness of speech and make the recording comfortable for young children. Instead, a sparkling golden star conforming to the experimental decoration was placed right above the camera helping the children to keep their head stable and look straight. Trials during which participants moved were discarded subsequent to the recordings via visual inspection of the video data.

Teams of two experimenters conducted the recordings. The first one familiarized the participant with the SOLLAR platform and introduced the children to the story the production task was embedded in. She maintained a face-to-face connection with the participant throughout the recording, controlled for head movement as well as correct pronunciation, and prompted the audio stimuli. The second experimenter operated SOLLAR’s recording equipment from a desk not visible to the participant. S/he controlled for the quality of the data collection by thoroughly monitoring both video and audio streams and interrupted if necessary.

The recording room was decorated in a universe theme allowing the experiment to be introduced to children as a space ship journey during which they had to repeat foreign words from other planets’ languages. This stimulated their interest and engagement in the task. Except for the chair, the setup was the same for children and adults, however, the adults were not introduced to the planet story.

### Data processing

The acoustic signal was recorded both in relation to the ultrasound device and the video camera, enabling the generation of a common time code for the three streams. A cross-correlation function within MATLAB [47] was used to synchronize the streams (cf. [7,48]).

Acoustic data served as a reference to define the relevant time points in the ultrasound signal. Therefore, target utterances and segments were first phonetically labeled using Praat [49]. For adults, the detection of target words and segments was done semi-automatically using WebMAUSBasic [50] and manual correction when necessary. For children, native speakers of German identified and manually labeled the target words for subsequent detection and manual labelling of the target segments. A stable periodic cycle in the oscillogram as well as a stable formant pattern, especially a clearly detectable second formant, were used as indices for vocalic segments. The first ascending zero-crossing in the oscillogram at the beginning of the periodicity was accordingly used as schwa and vowel onset, the first ascending zero-crossing after the end of periodicity and disappearance of F2 as the beginning of the following consonant. From the resulting intervals, the relevant time stamps for the current analysis, the temporal midpoint of the schwa and the temporal midpoint of the vowel were automatically extracted.

Repetitions that did not correspond to the model speaker’s word were discarded from further analysis, except for those of 3-year-olds. Here, the approach was to use as many correctly produced first syllables as possible, so words were kept as long as əC_1_V corresponded to the model speaker and C_2_ did not differ in place of articulation from the model word (e.g., /aɪnə ba:tə/ was kept for model /aɪnə ba:də/). This way, two instances of words with C_2_ = /k/ were kept for /g/, 17 with C_2_ = /t/ for /d/, and ten with C_2_ = /v/ for /b/.

Ultrasound frames of interest were selected based on the corresponding time stamps of the acoustic data. For each relevant frame, tongue contours were semi-automatically detected with scripts custom-made for MATLAB [47] as part of the SOLLAR platform. A spline was automatically fit to reference points that were manually placed on the visible midsagittal tongue surface contour for each frame individually. X- and y-coordinates for each of 100 points of these splines were automatically extracted (see S1 Fig for an illustration). For the present analyses, we used only the x-coordinate, hence the horizontal position, of the highest point of the tongue body surface contour as a representation of frontness of the tongue body.

### Data analysis

We used R [51] and lme4 [52] to investigate the three research questions. Our first research question addressed whether children in all age cohorts as well as adults anticipated the lingual position of the vowel during the preceding schwa. Because of previous evidence for the degree of V-to-V coarticulation to be modulated by the intervocalic consonant’s resistance, each consonant context was checked separately for each cohort. More specifically, we investigated whether the horizontal position of the highest point of the tongue body during the schwa midpoint (X_ə_) varied systematically depending on the position of the highest point of the tongue body during the vowel midpoint (X_V_).

To address this and the other two research questions, we fitted a linear mixed effects model regressing X_ə_ on X_V_, consonant context (Consonant1), and Cohort with their interactions. The random effect structure was selected following Bates and colleagues’ suggestions to use principal component analysis (PCA) for checking the dimensionality of the model and likelihood ratio tests for assessing its goodness of fit [53]. Starting from the full random effects structure by subject and word, smallest variance components were dropped step by step until convergence was reached and the PCA showed that the number of dimensions was supported by the data. This procedure resulted in a random effect structure including intercepts for subjects and words as well as by-subject random slopes for the effect of the consonant and by-word random slopes for the effect of cohort. The model’s assumptions were checked via visual inspection of residual plots and outliers were checked individually to either be removed (experimental errors) or corrected (processing errors). This did not change the outcome pattern.

The second research question focused on possible differences in V-to-V coarticulation degree between the three consonant contexts (/b, d, g/) within each cohort. We applied pairwise comparisons of the interactions between X_V_ and Consonant1 using generalized linear hypothesis tests with adjusted *p*-values (glht, multcomp package [54], *p*-value adjustment followed the truncated closed test procedure from Westfall [55]). All pairwise comparisons for the X_V_:Consonant1 interaction were obtained by manually setting the contrast matrix.

Finally, age-related developmental differences in coarticulation degree within the three consonant contexts were addressed using pairwise glht comparisons of the interactions between X_V_ and Cohort that were again obtained with a manually set contrast matrix using Westfall-adjusted *p*-values. Additionally, the three-way-interactions of X_V_, Cohort, and Consonant1 indicated whether the differences of the consonant contexts’ effects on coarticulation magnitude (i.e. the coarticulation pattern) vary with age cohort.

## Results

### Vowel-to-vowel coarticulation in every age cohort

**Fig 1:**
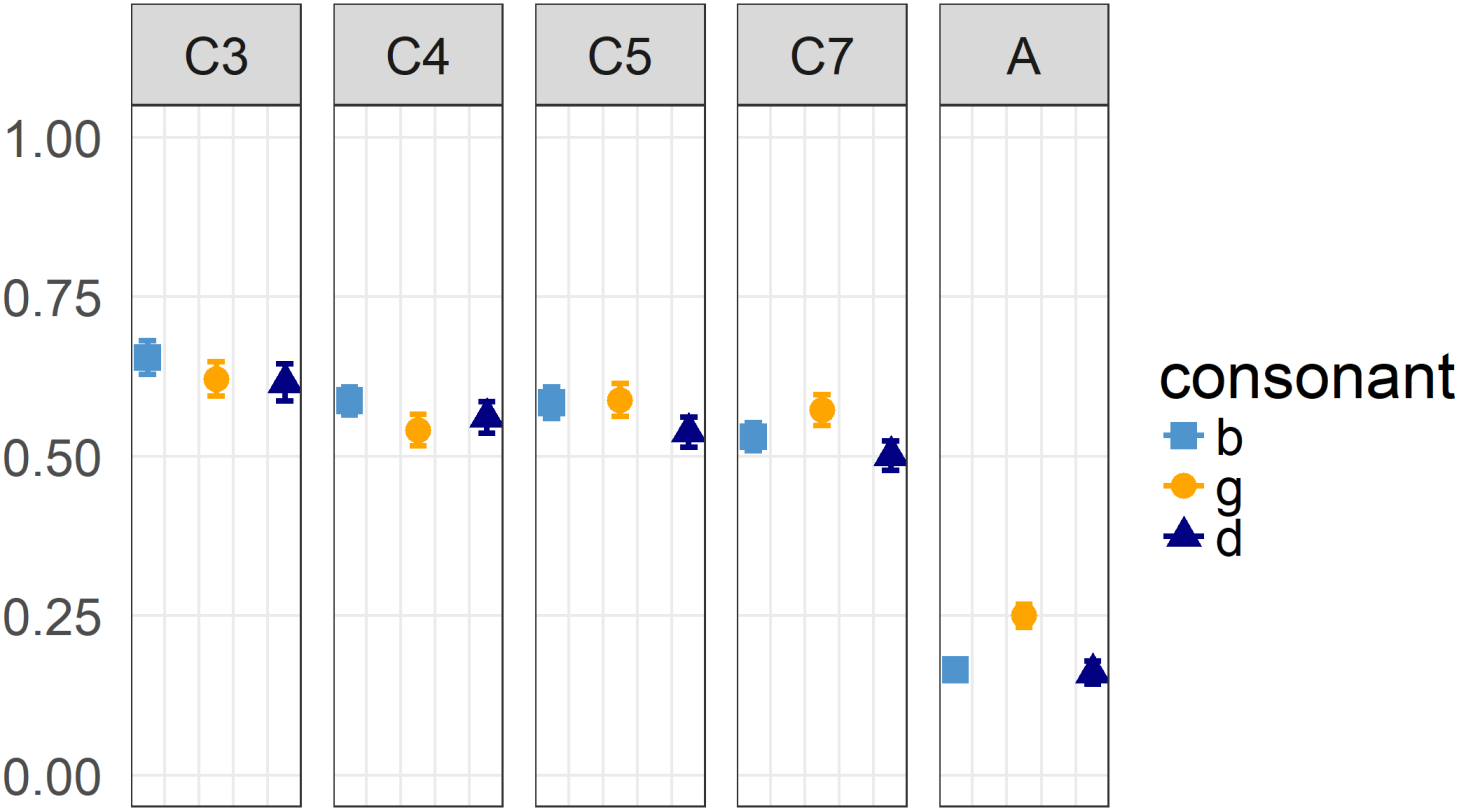
Regression coefficients for the three consonant contexts /b, d, g/ per cohort. Error bars represent one standard error of the coefficients.

The effect of the tongue’s horizontal position during the vowel on its position during the preceding schwa is significant for each consonant context in each age cohort (p < 0.001, see S2 Table for detailed model output). The coarticulation degree however, differs between the investigated age cohorts and consonant contexts as can be seen in the display of the regression coefficients (Fig 1). Statistical relevance of these differences will be addressed in the following sections.

### Consonantal impact only in adults

The results of the pairwise comparisons between the consonant contexts within each cohort are summarized in Table 1. The intervocalic consonant only has an effect on the V-to-V coarticulation degree in adults with əgV sequences allowing for more V-to-V coarticulation than both əbV and ədV sequences. In none of the cohorts of children does the nature of the intervocalic consonant significantly impact the degree of V-to-V coarticulation. There is only a trend (p = 0.0834) for /g/-contexts to allow for more coarticulation than /d/-contexts in 7-year-old children.

**Table 1:** Results of the linear hypotheses tests for consonantal differences within cohort.

Cohort	Hypothesis	Estimate	SE	z	*p*-value		*direction*
C3	b-d	0.039210	0.037024	1.059	0.539		
	b-g	0.033378	0.035412	0.943	0.613		
	d-g	-0.005832	0.038511	-0.151	0.987		
C4	b-d	0.02630	0.03214	0.818	0.691		
	b-g	0.04617	0.03162	1.460	0.310		
	d-g	0.01988	0.03453	0.576	0.833		
C5	b-d	0.046438	0.033473	1.387	0.347		
	b-g	-0.004137	0.034414	-0.120	0.992		
	d-g	-0.050574	0.034717	-1.457	0.312		
C7	b-d	0.02949	0.03115	0.947	0.6103		
	b-g	-0.04169	0.03166	-1.317	0.3855		
	d-g	-0.07118	0.03340	-2.131	0.0834	.	
A	b-d	0.00461	0.02317	0.199	0.97836		
	b-g	-0.08467	0.02345	-3.611	0.00087	***	*b < g*
	d-g	-0.08928	0.02565	-3.481	0.00135	**	*d < g*

Results were obtained via glht comparisons with Westfall *p*-value adjustment. Cohort abbreviations are C3—3-year-old children, C4—4-year-old children, C5—5-year-old children, C7—7-year-old children, and A—adults. The last column indicates the direction of significant effects. Significance codes '***': p< .001; '**': p< .01; '*': p< .05; '.': p< 0.1

### Developmental decrease of V-to-V coarticulation magnitude

To assess differences in coarticulation degree between the cohorts, first, pairwise glht comparisons of the X_V_:Cohort interactions were run (see Table 2). For every consonant context, the degree of V-to-V coarticulation is significantly lower in the adult cohort than in each of the cohorts of children. In the /b/ and /d/-contexts, there are additional statistically significant differences between the 3-year-olds and each of the 7-year-olds: The youngest participants show a higher degree of coarticulation from the vowel to the preceding schwa than the oldest cohort of children for əbV and ədV sequences

**Table 2:** Results of the linear hypotheses tests for cohort differences within consonant contexts.

Consonant	Hypothesis	Estimate	SE	z	*p*-value		*direction*
b	A—C3	-0.489537	0.030386	-16.111	<0.001	***	*A < C3*
	A—C4	-0.422136	0.026773	-15.767	<0.001	***	*A < C4*
	A—C5	-0.419119	0.026814	-15.631	<0.001	***	*A < C5*
	A—C7	-0.365634	0.024724	-14.789	<0.001	***	*A < C7*
	C7—C3	-0.123903	0.031742	-3.903	<0.001	***	*C7 < C3*
	C7—C4	-0.056502	0.026632	-2.122	0.208		
	C7—C5	-0.053484	0.027010	-1.980	0.273		
	C5—C3	-0.070419	0.032696	-2.154	0.196		
	C5—C4	-0.003018	0.029451	-0.102	1.000		
	C4—C3	-0.067401	0.030860	-2.184	0.184		
d	A—C3	-0.45494	0.03406	-13.357	<0.001	***	*A < C3*
	A—C4	-0.40045	0.03081	-12.998	<0.001	***	*A < C4*
	A—C5	-0.37729	0.02816	-13.396	<0.001	***	*A < C5*
	A—C7	-0.34075	0.02733	-12.469	<0.001	***	*A < C7*
	C7—C3	-0.11418	0.03531	-3.234	0.0105	*	*C7 < C3*
	C7—C4	-0.05970	0.03081	-1.937	0.2946		
	C7—C5	-0.03654	0.02898	-1.261	0.7127		
	C5—C3	-0.07765	0.03528	-2.201	0.1773		
	C5—C4	-0.02316	0.03237	-0.715	0.9524		
	C4—C3	-0.05449	0.03568	-1.527	0.5416		
g	A—C3	-0.37149	0.03250	-11.429	<0.001	***	*A < C3*
	A—C4	-0.29130	0.03063	-9.511	<0.001	***	*A < C4*
	A—C5	-0.33859	0.02939	-11.519	<0.001	***	*A < C5*
	A—C7	-0.32265	0.02823	-11.431	<0.001	***	*A < C7*
	C7—C3	-0.04884	0.03395	-1.439	0.601		
	C7—C4	0.03136	0.03047	1.029	0.841		
	C7—C5	-0.01593	0.02972	-0.536	0.983		
	C5—C3	-0.03290	0.03427	-0.960	0.872		
	C5—C4	0.04729	0.03245	1.457	0.589		
	C4—C3	-0.08020	0.03337	-2.403	0.113		

Results were obtained via glht comparisons with Westfall *p*-value adjustment. Cohort abbreviations are C3—3-year-old children, C4—4-year-old children, C5—5-year-old children, C7—7-year-old children, and A—adults. The last column indicates the direction of significant effects. Significance codes '***': p< .001; '**': p< .01; '*': p< .05; '.': p< 0.1

In a second step, coarticulatory patterns were compared between cohorts by running three-way-interactions of the effects of X_V_, Cohort, and Consonant1. Table 3 provides the model output for those interactions that reached significance or indicated a trend. The difference in V-to-V coarticulation degree between /b/- and /g/-contexts is different between adults and each of the three younger cohorts of children (3-, 4-, and 5-year-olds). It is also different between 7-year-olds and the two youngest age cohorts (only marginally significant between 3-year-olds and 7-year-olds). Fig 1 visualizes these differences: While for adults /g/-contexts allow for more coarticulation than /b/-contexts, the direction of the (non-significant) difference is the other way around for young children. Regarding the difference between /d/- and /g/-contexts, adults’ pattern only differs significantly from 4-year-olds’ with a trend in comparison to 3-year-olds. In addition, 4-year-olds differ from 7-year-olds.

**Table 3:** Summary of the significant and marginally significant three-way-interactions of the effects of X_V_, Cohort, and Consonant1.

Consonants	Cohorts	Estimate	SE	t value	*p*-value	
b / g	A / C3	0.118045	0.042239	2.795	0.005513	**
	A / C4	0.13084	0.03920	3.338	0.001026	**
	A / C5	0.08053	0.03874	2.079	0.037877	*
	C7 / C3	0.075065	0.044536	1.686	0.093453	.
	C7 / C4	0.087860	0.039424	2.229	0.026535	*
g / d	A / C3	0.08344	0.04610	1.810	0.071136	.
	A / C4	0.10915	0.04293	2.542	0.011679	*
	C7 / C4	0.09105	0.04298	2.118	0.0348	*

Cohort abbreviations are C3—3-year-old children, C4—4-year-old children, C5—5-year-old children, C7—7-year-old children, and A—adults. Significance codes '***': p< .001; '**': p< .01; '*': p< .05; '.': p< 0.1

## Discussion

Long-distance coarticulatory processes have been shown to provide valuable information about general speech production mechanisms. However, after Öhman’s work on lingual vowel-to-vowel coarticulation’s implications for principles of the speech production process [9], extensive investigations of the topic have been scarce. Similarly, while a substantial number of studies have compared children’s intrasyllabic coarticulation to adults’ (e.g., in the acoustic domain: [4,33]; in the articulatory domain: [7,32,37]), coarticulation beyond the syllabic frame has been the topic of only a handful of developmental studies so far. Yet, longer-distance coarticulatory processes can help to elucidate what aspects of (co)articulation may be planned while others may rather reflect byproducts of the gestures’ implementation in the vocal tract. From a developmental standpoint, this is a highly relevant question because it can shed light on the maturation of spoken language fluency and tease apart the factors that may impact this process.

The current study aimed to contribute to this endeavor by thoroughly investigating lingual vowel-to-vowel coarticulation in a larger participant pool of adults than previously examined as well as in four different age groups across childhood. In addition to testing for the presence of V-to-V coarticulation in each age cohort, we examined the potential impact of intervocalic consonants on the degree of V-to-V coarticulation. Most importantly, we compared coarticulatory patterns (both in terms of degree and consonantal impact) between age cohorts to unveil the maturation of these aspects of the speech production process. The discussion section is framed along these three main questions.

### Vocalic gesture’s anticipation

Results from this study provide strong evidence that adults anticipate a full vowel’s horizontal tongue position during a preceding schwa in əCV sequences. This finding extends previous research [9–19] with a larger sample of adult participants and provides insights into V-to-V coarticulatory patterns in German, a language whose coarticulation patterns have not been extensively investigated (e.g., [17,56]).

A second main finding is that all four cohorts of children exhibited strong vowel anticipation across syllable boundaries as well. This result is in line with the majority of studies addressing children’s V-to-V coarticulation [4,35,40–42] and augments previous evidence with data from German. However, this result conflicts with those of three existing studies. In particular, Repp [38] and Hodge [39] did not find any significant vocalic effect on the preceding schwa in 4- and 3-year-olds respectively, but only in their older participants ([38]: 9 years, [39]: 5 & 9 years). On the contrary, our data show that at 3.5 years of age, German children do anticipate the tongue body position for target vowels well ahead of their acoustic onsets. Note that Repp’s [38] results are based on a single speaker per age group only, which prevents strong conclusions. Hodge’s [39] sample size of 10 children per age cohort however, yields greater statistical power and generalizability. Yet, in contrast to other studies including ours, she used utterances containing /st/ clusters (“a stew” versus “a stee”) instead of singleton intervocalic consonants. It is well known that stable productions of consonant clusters are achieved relatively late in childhood (for a review, see [57]). For example, Smit and colleagues [58] reported that English-speaking children do not reach 75% production accuracy for /st/ clusters before the age of 4;06. Given this protracted maturation, studies addressing coarticulatory degree in sequences containing clusters and those testing singleton consonants are not directly comparable.

In a more recent study using ultrasound tongue imaging, Barbier and colleagues [45] reported neither acoustic nor articulatory evidence for V-to-V coarticulation in 4-year-old children. This strong contradiction with our finding may stem from substantial methodological differences between the two studies (e.g., V_1_ being a full vowel versus a schwa, using the whole tongue contour versus a point measure). Furthermore, the authors found a significant effect of vowel anticipation in the acoustic (effect of V_2_ on V_1_’s second formant) as well as in the articulatory data (vocalic anticipation in the front-back dimension) of some 4-year-old children. It is therefore surprising that they did not elaborate on these results but instead suggested an “inability to anticipate V_2_ in V_1_ during the production of V_1_-C-V_2_ sequences” for 4-year-olds (p. 4).

From our results, it is clear that like adults, children from at least 3.5 years of age anticipate the horizontal tongue position of a full vowel during the production of a preceding schwa across an intervocalic consonant. Whether this process should be interpreted as an “ability” or rather as an inevitable byproduct of continuous speech will be discussed in more detail in the following sections.

### The impact of the intervocalic consonant

In line with previous evidence [9,18,59] we found a significant impact of the consonant context on the degree of vocalic anticipation in adults: In əgV sequences, vowel anticipation was stronger than in əbV and in ədV sequences.

Both əgV and ədV are homorganic sequences because the tongue provides the primary articulators involved in the production of both consonantal and vocalic gestures. However, while the location of tongue body contact with the palate for /g/ is relatively flexible without affecting intelligibility, the contact point for /d/ is more constrained in the alveolar region [27]. In a previous investigation of intrasyllabic coarticulation in our cohort of adults, this strong difference in coarticulatory resistance between /g/ and /d/ was replicated [60]. The present finding of more vocalic anticipation in əgV than in ədV sequences is therefore neatly in line with the idea that the consonant’s resistance not only accounts for the degree of coarticulation during the consonant production but also for the degree of interference with transconsonantal coarticulation processes. Yet, if the consonant’s resistance were the only factor here, one would expect əbV sequences to exhibit the highest degree of lingual V-to-V coarticulation because the tongue body is not recruited for the labial occlusion gesture and can therefore anticipate the upcoming vowel’s gestures freely. Many studies including our previous analyses found the predicted high degree of lingual anticipation during /b/ in intrasyllabic coarticulation [2,18,25,26,37]. The present findings in intersyllabic coarticulation however, provide evidence for /b/ to allow V-to-V coarticulation (only) to the same extent as the high resistant /d/ instead of being very permeable for transconsonantal vowel anticipation as expectable for low resistant consonants like /b/ and /g/. A closer look at Fowler & Brancazio’s [18] data also reveals less V-to-V coarticulation in /b/ than in /g/ sequences for tongue fronting in one of two speakers and for F2 changes, both speakers exhibited less V-to-V coarticulation in /b/ contexts than in /g/ and /d/ contexts.

However, the origins of /b/’s and /g/’s low resistance are certainly distinct and must be acknowledged in order to understand their contrasting impact on V-to-V coarticulation: While /g/ engages the same primary articulator as following vowels (the tongue body), əbV sequences are heterorganic with /b/ not actively recruiting the tongue body. So, while for gV sequences, the position of the primary articulator, is changed by coproduction with the following vowel, gestural blending does not affect the primary articulator of /b/ (the lips) but an articulator that is not actively controlled for the production of /b/. Although both consonants are classified as low resistant because of the flexibility of the tongue body’s horizontal position, the sources of this high degree of coarticulation are thus very different in nature.

Looking only at the change of the tongue body’s position during the consonant, this difference results in more coarticulation during /b/ than during /g/ because an unspecified or inactive articulator can be changed most flexibly. However, in long-distance processes like vowel-to-vowel coarticulation across these consonants, the picture changes: The primary articulator of the consonant must start moving towards its target during the schwa to ensure the correct place of contact. For /g/ this means that during schwa, the tongue body moves towards a position in the velar or palatal region that will be more front in the case of following front vowels or back for following back vowels. The process of vowel-to-vowel coarticulation in əgV sequences could therefore be understood as being reinforced by /g/: because of the coproduction with the vocalic gesture, the contact point of the tongue body and the palate or velum is changed for /g/; In addition to the direct vocalic anticipation, the initiation of /g/ therefore increases the strength towards a front or back positioning of the tongue body during schwa. Yet, for /b/ the primary articulators are the lips, so they are the ones starting to move towards each other during schwa in a əbV sequence. There is no consonant-induced need however for the tongue back to start moving towards a specific position because it is unspecified for /b/. While during /b/ the vowel’s tongue position is thus anticipated, there is only a weaker vowel-related movement of the tongue towards that target during schwa.

Turning to children, in none of the investigated age groups did the nature of the intervocalic consonant influence the degree of V-to-V coarticulation significantly. There is only a marginally significant trend for 7-year-olds to coarticulate more in /g/- than in /d/-contexts similar to adults (*p* = 0.0834). Because previous developmental research has not focused on consonantal effects on vowel anticipation, the lack of consonantal impact is an important finding, especially given the sizeable difference found in comparison to adults. Although Nittrouer [40] examined the intervocalic consonant’s effect on V-to-V coarticulation in her data set of 3-, 5-, and 7-year old children and adults and found stronger vowel anticipation in /k/ compared to /t/ contexts, her study was not designed to address developmental differences of this effect. The age-related differences in the consonant’s impact that we found in our study as well as its implications for our understanding of the development of spoken language fluency will be discussed in the following section.

### Developmental differences

The overarching aim of the present study was to investigate the development of intersyllabic V-to-V lingual coarticulation. Expanding on our earlier findings regarding the organization of intrasyllabic V-to-C-coarticulation [37], the present results provide strong evidence for children to exhibit a much higher degree of V-to-V coarticulation than adults (cf., Fig 1). Children therefore seem to exhibit a larger extent of gestural overlap not only between the consonant and the following vowel but also earlier during the preconsonantal schwa. This suggests that children initiate vocalic gestures earlier in comparison to adults. Kent [34] described developing (as well as impaired) speech production to follow a principle of “everything moves at once” (p.70). A conceivable reason for this greater gestural overlap in children compared to adults might be the lack of inhibitory control that is well attested in various cognitive domains for young children (e.g., [61]). A lower inhibition level might accordingly lead to more simultaneously activated gestures and hence more articulatory overlap (cf. [62]).

Among the different cohorts of children, we also noticed a trend towards a developmental decrease in coarticulation degree from 3.5 to 7 years of age, but it only yields significance for the alveolar and bilabial context, not for the velar one. Indeed, for sequences involving the resistant consonant /d/ the youngest group of children at 3.5 years of age exhibits significantly more coarticulation than the oldest group. Both the alveolar stop /d/ and the bilabial stop /b/ requires a very fine spatiotemporal coordination of different articulators: The tongue’s subparts (e.g., the tongue tip and the tongue body) in ədV sequences, the lips and the tongue body in əbV sequences. Whether a maturation of this coordination between 3 and 7 years of age is the reason for our preliminary finding should be investigated more thoroughly with a larger set of consonants requiring fine lingual coordination (e.g., /d, t, z, s, l, n, f, w, m, n/). Yet, this result accords well with previous reports on the non-uniform development of articulatory controls for speech (lips and jaw: e.g., [63,64]; for the tongue: e.g., [7,65]). It further suggests that the developmental spurts and plateaus often reported for other articulators in the literature (e.g., great change in lip movements variability between 2 and 6 years [66], variability plateau between 7 and 12 years [64]) should be carefully interpreted in relation to the speech material investigated and the complexity of the gestural coordination involved. In practice, the differences in V-to-V coarticulatory degree within childhood certainly call for more scrupulous investigations of coarticulatory patterns in tightly clustered age groups. Such research would provide a description of gestural control development across childhood preventing important transitions from remaining unnoticed. It would further provide much needed normative data to disentangle coarticulatory differences that reflect typical trajectories from those that may predict later articulatory disfluencies. This may for instance be particularly relevant for the early assessment of children with developmental apraxia of speech known to show impairments of speech motor control (see review in [67]).

Interestingly, the developments of V-to-C and V-to-V coarticulation do not seem to go uniformly hand in hand. While the present study unveiled a change in V-to-V coarticulation degree during childhood only between the youngest and the oldest children for sequences involving the alveolar stop /d/ and the bilabial /b/, our earlier results on V-to-C coarticulation provided evidence for significant differences between cohorts of children for /b/ (C3 > C7) and /g/ (C3 > C7, C5 > C7) but not for /d/ [37]. This finding again highlights the very different role the articulatory properties of a consonant play for inter- and intrasyllabic coarticulation processes outlined above.

In our study, the gap in the magnitude of coarticulation between 7-year-olds and adults remains tremendous across all consonants. Children at the beginning of primary school are therefore still developing the organization of lingual gestures for articulatory fluency. This result supports previous research pointing at the protracted development of spatiotemporal control of speech gestures [64]. Despite an increasing interest for addressing early language development in recent years, future research should include late childhood investigations to locate transitions towards adult-like patterns of coarticulation and identify the factors responsible for developmental differences across childhood. Note that the nature of those factors may change over time. While age-related differences might initially be driven by discrepancies in lexical knowledge (e.g., [68,69]) and/or speech motor control, coarticulatory differences between older groups of children may be affected by the acquisition of new skills (e.g., inhibitory control [62]) or consolidation of recently acquired ones.

The second developmental difference found in our data is that the consonant’s identity impacts on the degree of V-to-V coarticulation in adults but (except for a marginally significant trend for 7-year-olds) not in children. While we found adult-like patterns of consonants’ coarticulatory resistance in our previous analyses on children’s intrasyllabic coarticulation [37], the strong discrepancy between ages in intersyllabic coarticulation seems surprising at first glance. However, as predicted, adults’ effects of the consonant’s identity seem to be stronger in intra- than in intersyllabic coarticulation. Being relatively subtle, consonantal effects might therefore be concealed by the higher variability in children’s intersyllabic coarticulation. Taking the conducted three-way-interactions of the factors X_V_, Cohort, and Consonant1 into account, it becomes obvious however, that the different behavior of adults and children cannot solely result from too high variability: The /g/>/b, d/ pattern observed in adults seems only to develop across the investigated age cohorts. While for 3- and 4-year-olds both the relations between the /b/- and /g/-context and that between the /d/- and /g/-context differ from that of adults, it is only the b-g relation that is different between 5-year-olds and adults. 7-year-olds pattern in the same way as adults. Albeit the coarticulation degree of 7-year-olds is still very different from that observed in adults, the coarticulatory pattern regarding the relation of /b/, /d/, and /g/-contexts therefore is already approximating that of adults.

Linking these findings, we see hints for the hypothesis that the diverging roles of the intervocalic consonant in children’s and adults’ V-to-V coarticulation is based in their gestural organization. As Öhman [9] and Fowler & Brancazio [18], argued, vocalic gestures may be phased relatively invariantly with each other while the consonantal gesture occurs as a temporally limited event during the broad vocalic movements. Given that young German children were reported to focus on stressed syllables [70] and that due to their acoustic and prosodic properties vowels seem to have a special status in an utterance, functioning as attractors and being very prominent for children [71], articulatory gestures relating to V_2_ (the stressed full vowel in our stimuli) might be hardest to inhibit for children and therefore show especially broad overlap with preceding gestures. Any subtle effect of the intervocalic consonant’s resistance might therefore not (only) be concealed by a high variability but by an underlying outstandingly high prominence of vowels in child speech. In contrast to the idea of consonant-mediated V-to-V effects in adult speech, V-to-V coarticulation in children would therefore be interpreted as a pure coproduction of V_1_ and V_2_ because of the greater gestural overlap. Accordingly, while the V-to-V coarticulation degree in velar contexts is especially high presumably because of the stop /g/ mediating coarticulation, this context does not promote stronger coarticulation than the others in young children’s speech. The strong coproduction of V_1_ and V_2_ itself, results in approximately the same coarticulation degree in all contexts.

Another factor possibly influencing the maturation of coarticulatory processes during childhood is the anatomical development of the vocal tract. While it is well known that physiological characteristics can affect articulation (e.g. [72,73]), evaluating the precise impact of those anatomical changes on developmental differences in lingual coarticulation remains an empirical challenge. To overcome difficulties in anatomical measurements of the vocal tract or the tongue, the growing research on articulatory modelling may provide better estimates (e.g., [74,75]).

### Limitations and perspectives

Investigating lingual coarticulation in the first years of life has become increasingly significant for the early detection of spoken language deviancies. However, collecting quantitative tongue data from young children is not exempt of methodological challenges (e.g., limited attention span, intolerance to invasive methods and too long data collection sessions). In this study as in previous research, a few compromises were therefore necessary to meet our research goals.

First, we used a customized probe holder designed to not impede natural jaw movements and collect data that approximate natural speech conditions more faithfully than if we had used a helmet (e.g., [32]). We employed three strategies to prevent head movement artefacts: 1) the SOLLAR recording platform included a car seat with seatbelts; 2) a bright star was positioned in front of the child as a visual fixation point; 3) one of the experimenters sat in front of the child to maintain visual contact and monitor the child’s position. Finally, post-recording examination using video data was conducted to discard data in which children moved.

Second, following up on previous research (e.g.,[7,37]), this study employed measurements of the highest point on the tongue body to assess variation in the gestural organization of V-to-V coarticulation. While the approach to use a single point measure is certainly convenient for the investigation of large samples, it is not optimal for fine-grained distinctions between the subparts of the tongue (e.g., tongue root) as in studies considering the full tongue contour (e.g., [21]). However, it is important to acknowledge that the reliability of the latter approach highly depends on the quality of the tongue imaging at the two ends of the tongue contour (cf. [37] for a more detailed discussion). In previous studies the measure employed here has provided meaningful results as to developmental differences in coarticulatory overlap (e.g., [7,26,37]). Most acoustic studies used measurements of F2 as an estimate of the tongue position along the antero-posterior dimension and the resulting cavities (e.g., adults: [9–13], children: [4,35,38–42]). The highest point on the tongue body is the most salient for vowel constriction and therefore provides a more direct access to those parameters. Future studies of lingual coarticulation will gain in designing methodologies that integrate measurements of fixed point parameters and of the full tongue contour. With such a combinatorial approach, it will be possible to unveil subtle developmental differences in coarticulatory patterning, due for instance to discrepancies in coordinative control of the tongue’s functional subparts.

Finally, we are well aware that assessing vocalic anticipation via single time point analyses is not optimal because the method does not fully capture coarticulation dynamics (e.g., [76]). The optimization of analytical approaches assessing change over time to ultrasound research will be necessary to unveil the complexity of gestural dynamics (e.g., [77]).

## Conclusion

This study was the first which addressed the maturation of lingual long-distance coarticulatory processes in a cross-sectional investigation of five age cohorts using articulatory measurements. Taken together, our findings provide evidence for children to exhibit stronger vocalic anticipation than adults suggesting a maturational decrease of gestural overlap with age. Across the period from 3.5 to 7 years of age, no general, but a consonant context-specific decrease of vocalic anticipation was found, which is a sign of non-uniform maturation of gestural organization possibly driven by differences in articulatory complexity. The tremendous disparity in coarticulation degree between the oldest children investigated and the adults indicates that the development of adult-like gestural organization continues during late childhood. Our study therefore highlights the importance of investigations of older children’s and adolescents’ speech to uncover factors that might lead to a compression of articulatory gestures, hence an adult-like lower gestural overlap.

## Supporting information

S1 Table Summary of the number of analyzed trials per consonant context per age cohort. Cohort abbreviations are C3–3-year-old children, C4–4-year-old children, C5–5-year-old children, C7–7-year-old children, and A—adults.
(DOCX)

S2 Table Model output for the vowel’s effect on schwa in every consonant context for each cohort. Cohort abbreviations are C3–3-year-old children, C4–4-year-old children, C5–5-year-old children, C7–7-year-old children, and A—adults.
(DOCX)

S1 Fig Ultrasound data. Raw ultrasound image of a 5-year-old boy’s tongue (CM5_005) at the temporal midpoint of the articulation of an [e] on the left and the semi-automatically labeled surface contour on top of the same frame on the right side. The tip of the tongue is to the left in both images.
(DOCX)

S1 Dataset Measures of the horizontal position of the highest point on the tongue dorsum. Column description: SUBJECT—Participant ID (Cohort abbreviation, F—female, M—male, T (in 7-year-olds only: typically developing)), BLOCK—Number of block the word was recorded in, TRIAL—trial number, WORD—stimulus word, CONSONANT1 –C_1_ in /aɪnə C_1_VC_2_ə/, CONSONANT2 –C_2_ in /aɪnə C_1_VC_2_ə/, VOWEL—V in /aɪnə C_1_VC_2_ə/, CV_PAIR—C_1_V in /aɪnə C_1_VC_2_ə/, PEAKX_a1_50 –horizontal position of the highest point on the tongue dorsum at the temporal midpoint of ə in /aɪnə/, PEAKX_V50—horizontal position of the highest point on the tongue dorsum at the temporal midpoint of V in /aɪnə C_1_VC_2_ə/, COHORT—Cohort abbreviation (A—adults, C3–3-year-old children, C4–4-year-old children, C5–5-year-old children, G1–7-year-old (‘grade one’) children).
(CSV)
