# Supplementary material for: On the development of gestural organization: A cross-sectional study of vowel-to-vowel anticipatory coarticulation
Source: PLoS One. 2018 Sep 14;13(9):e0203562. doi: 10.1371/journal.pone.0203562 (PMC6138403; doi:10.1371/journal.pone.0203562)
Supplement: S1 Table — Cohort abbreviations are C3–3-year-old children, C4–4-year-old children, C5–5-year-old children, C7–7-year-old children, and A—adults. (DOCX) [file pone.0203562.s001.docx]

**S1 Table. Summary of the number of analyzed trials per consonant context per age cohort.**

| **Cohort** | **Consonant Context** | **Number of trials** |
| --- | --- | --- |
| C3 | əbV | 534 |
|  | ədV | 517 |
|  | əgV | 485 |
| C4 | əbV | 552 |
|  | ədV | 566 |
|  | əgV | 551 |
| C5 | əbV | 522 |
|  | ədV | 545 |
|  | əgV | 509 |
| C7 | əbV | 638 |
|  | ədV | 655 |
|  | əgV | 653 |
| A | əbV | 723 |
|  | ədV | 723 |
|  | əgV | 722 |

Cohort abbreviations are C3 – 3-year-old children, C4 – 4-year-old children, C5 – 5-year-old children, C7 – 7-year-old children, and A – adults.
