# Supplementary material for: On the development of gestural organization: A cross-sectional study of vowel-to-vowel anticipatory coarticulation
Source: PLoS One. 2018 Sep 14;13(9):e0203562. doi: 10.1371/journal.pone.0203562 (PMC6138403; doi:10.1371/journal.pone.0203562)
Supplement: S2 Table — Cohort abbreviations are C3–3-year-old children, C4–4-year-old children, C5–5-year-old children, C7–7-year-old children, and A—adults. (DOCX) [file pone.0203562.s002.docx]

**S2 Table. Model output for the vowel’s effect on schwa in every consonant context for each cohort.**

| **Cohort** | **Consonant** | **β-coefficient** | **SE** | **t-value** | ***p*-value** | |
| --- | --- | --- | --- | --- | --- | --- |
| C3 | b | 0.654484 | 0.026293 | 24.892 | <0.001 | *** |
|  | d | 0.615274 | 0.029026 | 21.197 | <0.001 | *** |
|  | g | 0.621107 | 0.026886 | 23.101 | <0.001 | *** |
| C4 | b | 0.587084 | 0.021988 | 26.700 | <0.001 | *** |
|  | d | 0.560788 | 0.025054 | 22.384 | <0.001 | *** |
|  | g | 0.54091 | 0.02448 | 22.094 | <0.001 | *** |
| C5 | b | 0.584066 | 0.024605 | 23.738 | <0.001 | *** |
|  | d | 0.537628 | 0.023936 | 22.461 | <0.001 | *** |
|  | g | 0.588202 | 0.025618 | 22.961 | <0.001 | *** |
| C7 | b | 0.530582 | 0.022336 | 23.755 | <0.001 | *** |
|  | d | 0.501092 | 0.023329 | 21.480 | <0.001 | *** |
|  | g | 0.57227 | 0.02453 | 23.332 | <0.001 | *** |
| A | b | 0.16495 | 0.01577 | 10.462 | <0.001 | *** |
|  | d | 0.16034 | 0.01794 | 8.939 | <0.001 | *** |
|  | g | 0.24961 | 0.01868 | 13.361 | <0.001 | *** |

Cohort abbreviations are C3 – 3-year-old children, C4 – 4-year-old children, C5 – 5-year-old children, C7 – 7-year-old children, and A – adults.
