# Supplementary material for: On the development of gestural organization: A cross-sectional study of vowel-to-vowel anticipatory coarticulation
Source: PLoS One. 2018 Sep 14;13(9):e0203562. doi: 10.1371/journal.pone.0203562 (PMC6138403; doi:10.1371/journal.pone.0203562)
Supplement: S1 Fig — Raw ultrasound image of a 5-year-old boy’s tongue (CM5_005) at the temporal midpoint of the articulation of an [e] on the left and the semi-automatically labeled surface contour on top of the same frame on the right side. The tip of the tongue is to the left in both images. (DOCX) [file pone.0203562.s003.docx]

**S1 Figure. Ultrasound data.**


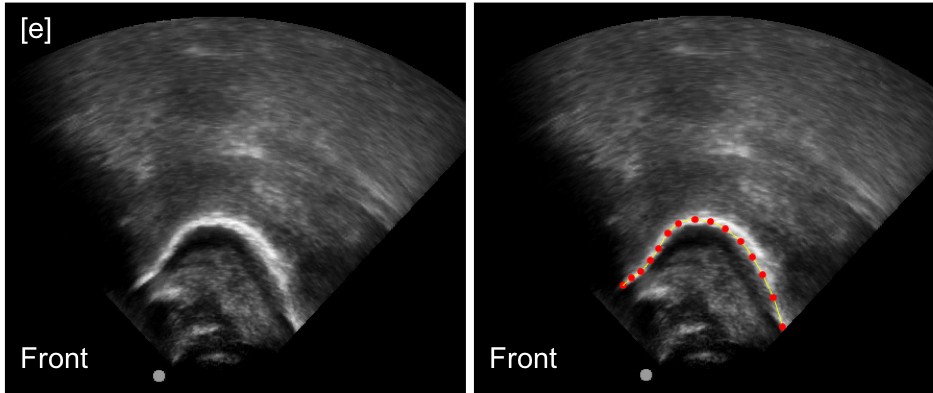
Raw ultrasound image of a 5-year-old boy’s tongue (CM5_005) at the temporal midpoint of the articulation of an [e] on the left and the semi-automatically labeled surface contour on top of the same frame on the right side. The tip of the tongue is to the left in both images.
